# Low Incidence of Chest Wall Pain with a Risk-Adapted Lung Stereotactic Body Radiation Therapy Approach Using Three or Five Fractions Based on Chest Wall Dosimetry

**DOI:** 10.1371/journal.pone.0094859

**Published:** 2014-04-11

**Authors:** Thibaud P. Coroller, Raymond H. Mak, John H. Lewis, Elizabeth H. Baldini, Aileen B. Chen, Yolonda L. Colson, Fred L. Hacker, Gretchen Hermann, David Kozono, Edward Mannarino, Christina Molodowitch, Jon O. Wee, David J. Sher, Joseph H. Killoran

**Affiliations:** 1 Department of Radiation Oncology, Dana-Farber Cancer Institute/Brigham and Women's Hospital, Boston, Massachusetts, United States of America; 2 Department of Radiation Oncology, Harvard Medical School, Boston, Massachusetts, United States of America; 3 Division of Thoracic Surgery, Brigham and Women's Hospital, Boston, Massachusetts, United States of America; 4 Department of Radiation Oncology, Rush University Medical Center, Chicago, Illinois, United States of America; 5 Joseph Fourier University, Department of Engineering for Health and Medicine, Grenoble, France; University of Wisconsin School of Medicine and Public Health, United States of America

## Abstract

**Purpose:**

To examine the frequency and potential of dose-volume predictors for chest wall (CW) toxicity (pain and/or rib fracture) for patients receiving lung stereotactic body radiotherapy (SBRT) using treatment planning methods to minimize CW dose and a risk-adapted fractionation scheme.

**Methods:**

We reviewed data from 72 treatment plans, from 69 lung SBRT patients with at least one year of follow-up or CW toxicity, who were treated at our center between 2010 and 2013. Treatment plans were optimized to reduce CW dose and patients received a risk-adapted fractionation of 18 Gy×3 fractions (54 Gy total) if the CW V30 was less than 30 mL or 10–12 Gy×5 fractions (50–60 Gy total) otherwise. The association between CW toxicity and patient characteristics, treatment parameters and dose metrics, including biologically equivalent dose, were analyzed using logistic regression.

**Results:**

With a median follow-up of 20 months, 6 (8.3%) patients developed CW pain including three (4.2%) grade 1, two (2.8%) grade 2 and one (1.4%) grade 3. Five (6.9%) patients developed rib fractures, one of which was symptomatic. No significant associations between CW toxicity and patient and dosimetric variables were identified on univariate nor multivariate analysis.

**Conclusions:**

Optimization of treatment plans to reduce CW dose and a risk-adapted fractionation strategy of three or five fractions based on the CW V30 resulted in a low incidence of CW toxicity. Under these conditions, none of the patient characteristics or dose metrics we examined appeared to be predictive of CW pain.

## Introduction

Stereotactic body radiation therapy (SBRT) has emerged as an excellent alternative to surgical resection for patients with medically inoperable early stage non-small cell lung cancer (NSCLC) or oligometastatic lesions to the lung. SBRT is a treatment technique that delivers highly conformal, high dose radiation, with a hypofractionated scheme (e.g. 18 Gray×3 fractions), which is drastically different from conventionally fractionated radiation therapy (RT) which employs daily doses of ∼2 Gray per fraction delivered over several weeks.

Numerous single-institutional series [1]–[3] and phase II studies [4]–[6], including a multi-institutional Radiation Therapy Oncology Group (RTOG) study, have demonstrated high local control rates of greater than 80–90% with SBRT for lung tumors and a low risk of severe toxicity (<10%) when patients are appropriately selected.

However, early reports of lung SBRT demonstrated unique toxicity events that have not been previously seen with conventionally fractionated thoracic RT. In particular, chest wall (CW) toxicity, which typically occurs several months after SBRT, has been observed in multiple series with varying incidences [7]–[16]. CW toxicity includes a spectrum of clinical findings including rib fracture (symptomatic or asymptomatic), CW pain (focal or diffuse) or skin changes (erythema, ulceration). These early series evaluating SBRT toxicity defined the CW as an organ at risk (OAR).

One of the earliest CW toxicity publications, Dunlap et al [7], determined that the volume of CW receiving at least 30 Gy (V30) was associated with CW toxicity and that maintaining the CW (CW) V30 below 30 mL resulted in a trend toward reduced toxicity. Similarly, a series from Stephans et al [8] demonstrated a correlation between tumor size and CW dosimetry with CW toxicity and concluded that maintaining two dosimetric parameters for the CW, V30<30 mL and V60<3 mL, resulted in a lower (10–15%) risk of CW toxicity. In addition, Woody et al [9] employed the concept of modified equivalent dose (mEUD) [17] and concluded for their series, that mEUD was a better predictor of CW pain than V30.

Based on these early reports of CW toxicity, starting in 2010 we modified our departmental lung SBRT regimen to a risk-adapted fractionation approach [10], based primarily on the CW V30. With the goal of significantly reducing CW toxicity after treatment, peripheral tumors with minimal CW contact and CW V30≤30 ml were treated with 18 Gy×3 fractions (54 Gy total) while peripheral tumors with broad CW contact and/or CW V30>30 mL were treated with 10–12 Gy×5 fractions (50–60 Gy total).

In this study, we report the incidence of CW toxicity for patients treated with a risk-adapted SBRT approach based on CW dosimetry (V30). Also, we present a comprehensive analysis to identify other metrics that might further predict CW toxicity in these patients.

## Methods and Materials

### Patient selection and evaluation

This study was conducted with the approval of the institutional review board (IRB) for the Dana-Farber/Brigham and Women's cancer center. Because our study was a retrospective review of existing medical records, the requirement for a formal informed consent was waived by the IRB. Data used was de-identified to protect patient confidentiality.

Between the inception of our lung SBRT program in 2009 and 2013, 167 patients were treated at Brigham and Women's Hospital/Dana-Farber Cancer Institute for peripheral lung tumors using SBRT. From this initial data set, patients having a minimum of one year of follow-up or a CW toxicity event were included. The study cohort is comprised of 72 treatment plans for 69 patients. All patients in our study were treated with either 3 or 5 fractions of SBRT.

Patient clinical factors analyzed included age, gender, race, body mass index (BMI), diabetes and smoking history. Tumor factors included planning target volume (PTV), distance from PTV to CW, tumor stage and histology. Smoking status was categorized as: 1) never smokers; <100 cigarettes in their lifetime; 2) former smokers; quit smoking >1 year prior to diagnosis, and 3) current smokers; smoking at the time of diagnosis or had quit <1 year prior. Treatment factors included the number of fractions and the dose delivered to the CW determined from the CW Dose Volume Histogram (see below).

### Endpoints

As Shown in **Table 1**, CW toxicity was defined by the Common Terminology Criteria for Adverse Events (CTCAE) version 4.0, with the following modifications. Grade 1: mild pain requiring no pain medications, Grade 2: moderate pain requiring non-narcotic analgesics, and Grade 3: severe pain requiring narcotic analgesics. Patients whose toxicity level changed over time were scored at the time of greatest toxicity. For the three patients who received two separate courses of SBRT, the CW toxicity was scored separately for each lesion because the tumors were located in separate lobes.

**Table 1:** CTCAEv.4 chest wall pain toxicity scale and modified chest wall toxicity scale used in the current report.

Toxicity Grade	CTCAEv.4*	Modified Chest Wall Toxicity Assessment for Current Study
Grade 0	No pain	No pain
Grade 1	Mild pain	Mild pain, requiring no pain medicines
Grade 2	Moderate pain; limiting instrumental ADL	Moderate pain, requiring non-narcotic analgesics
Grade 3	Severe pain; limiting self care ADL	Severe pain, requiring narcotic analgesics

Primary tumor control and local control were defined as the absence of primary tumor or local failure, respectively, based on the definitions outlined in RTOG 0236. In brief, primary tumor failure was defined as (1) local enlargement defined as at least a 20% increase in the longest diameter of the gross tumor volume per CT scan and (2) evidence of tumor viability (either PET-CT demonstrating FDG-uptake of similar intensity as the pretreatment staging PET, or with pathologic confirmation via biopsy). Primary tumor failure included marginal failures occurring within 1 cm of the planning target volume (1.5–2.0 cm from the gross tumor volume). Failure beyond the primary tumor but within the involved lobe was also ascertained and local failure was defined as any primary tumor and/or involved lobe failure.

### SBRT Treatment and Follow-up Procedure

All patients were treated with risk-adapted SBRT per institutional norms, which included 1) restriction of SBRT to treat peripheral tumors only; 2) immobilization of patients with a vac-lock bag in a stereotactic frame; 3) use of abdominal compression to restrict tumor motion to <1 cm; 4) 4D-CT planning with generation of an internal target volume (ITV) using maximum-intensity projection and 4D cine; 5) use of a 5 mm PTV margin with no clinical target volume (CTV) margin; 6) prescribed dose using a risk-adapted approach of 11–12 Gy×5 fractions for tumors either in broad contact with the CW and/or with V30>30 mL, and 18 Gy×3 fractions for all other tumors; 7) treatment delivered every other day with daily image-guidance using ExacTrac®, cone-beam CT, and portal imaging. Treatment planning for all patients was performed using the Eclipse Treatment Planning system (Varian Medical Systems, Palo Alto, CA) and the AAA dose algorithm with heterogeneity corrections. The ITV was expanded by 5 mm volumetrically to create the PTV, but constrained from expanding into the CW. The CW was treated as an organ at risk during plan development with particular effort made to maintain V30<30 mL. Treatment planning methods such as beam angle selection and weighting were employed to minimize CW dose without compromising target coverage or violating constraints on other critical organs.

Patients were seen by a physician daily during treatment and then post-treatment, for a full history with attention to toxicity assessment including CW injury and physical exam, every 3–4 months for the first two years, then every 6 months for the next two years, and annually thereafter. Patients also underwent chest CT scans on the same schedule. These were used to assess the presence of rib fractures.

### Evaluation of the chest wall dose

**Figure 1:**
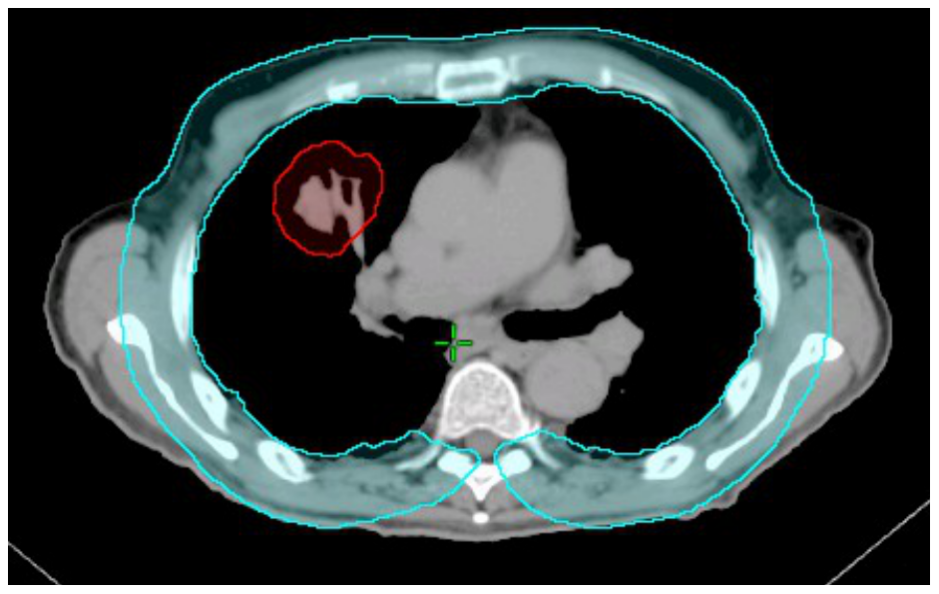
Chest wall and planning target volume (PTV) contour, in blue and red respectively.

The CW was contoured by first expanding the lungs uniformly by 3 cm and then subtracting lung volumes from the expanded volume. The CW volume was then edited to remove the mediastinum and spinal column. The remaining volume was defined as the CW volume as shown in **Figure 1**.

### Dose Metrics used for Statistical Analysis

We analyzed dose-volume metrics to identify predictors of CW toxicity, including maximum CW dose, (CW_max_), the minimum dose delivered to the 2 cc of CW receiving the highest dose (D2cc). Vx is the absolute volume of CW receiving a specified dose or higher (V20, V30, etc… where the number after the ‘V’ is the dose in Gy and the value is the volume in mL). e.g. V30 = 15 mL would mean that 15 mL of the CW is receiving at least 30 Gy. These values were tabulated for our analysis at dose levels from 20 to 70 Gy in 10 Gy increments.

Another dose metric we examined is the area under the curve (AUC) of the cumulative DVH curve. This was calculated by means of a simple integration routine for portions of the DVH above a threshold dose level, as well as for the entire DVH curve (threshold of zero). The AUC was calculated for threshold levels from 0 to 70 Gy in 10 Gy increments. The principle rationale behind the threshold value is that CW toxicity may only take effect above some minimum dose level. The AUC metric provides a broad measure roughly proportional to the integral dose, above the threshold level, received by the organ.

Finally, to account for the differences in biological effectiveness of the different fractionation schemes we calculated the modified equivalent dose (mEUD) using in-house developed software. As previously described by Woody *et al.*, the mEUD was calculated for a truncated [9] (i.e. “limited”) CW volume containing the 100 mL of tissue receiving the highest dose, which may be more relevant to the incidence of toxicity since only a small portion of the CW receives high dose.

The equation for mEUD is given by: 
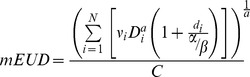



Where N is the total number of dose bins, 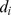 is the fractional dose and 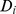 is the total prescribed dose for sub-volume 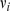. The dose 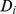 and corresponding fractional sub-volume 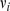 are readily determined from the differential form of the dose volume histogram. The fractional dose 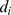 is simply 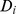 divided by the number of fractions and α and β are the linear and quadratic terms of the tissue-specific survival parameter, respectively. We used an α/β ratio of 3Gy for the CW. C is a normalization factor used to scale the result to be more easily comparable to a familiar fractionation scheme. This is used because mEUD, like biologically effective dose (BED), tends to inflate the resulting dose value when typical parameters are used. The normalization factor C produces a mEUD comparable to clinically understood dose levels [17]. The parameter ‘a’ is a tissue dependent parameter related to the Lyman model [18] parameter ‘n’ by a = 1/n. This parameter determines the degree to which the organ responds to radiation in a ‘parallel’ or ‘serial’ manner. For a completely parallel tissue a = 1 and it approaches positive infinity for completely serial tissue. When a = 1, the mEUD becomes the mean dose over the entire volume. Greater values of ‘*a*’ will place more importance on the higher doses. Since the value of ‘*a*’ does not have a definitive value for the CW, mEUD was calculated for a range of values of ‘*a*’ (a = 1 & a = 2.5–20 in steps of 2.5).

### Statistical analysis

We performed univariate logistic regression to test for associations between clinical patient clinical factors, tumor characteristics and dosimetry metrics with the endpoints of CW pain, rib fracture and a composite endpoint of CW pain or rib fracture. Multivariate analysis was also performed using a forward selection process with a threshold of inclusion in the model of p = 0.05. The Kaplan-Meier method was utilized to estimate the 1 and 2-year estimates of CW toxicity, primary tumor control, and local control, and median time to events. A *p-value* of 0.05 or less was considered to be statistically significant. Data were analyzed with Minitab 16.2.3 statistical software (Minitab Inc.) and SAS (SAS Institute Inc.).

## Results

### Patient and treatment characteristics

A summary of basic patient and treatment characteristics for the 72 treatment plans from 69 patients included in our study is shown in **Table 2**. The median patient age was 76 years (range 45–88). 58 (80.5%) patients were treated with conformal treatment and 14 (19.5%) using volume modulated arc therapy (VMAT).

**Table 2:** Patient and Treatment Characteristics for 72 peripheral lung SBRT treatment plans.

Characteristics	N (n = 72)	%	Median	Range
Age			76	45–88
**Gender**				
Male	31	41%		
Female	41	56.9%		
**PS**				
0–1	47	65.3%		
2	15	20.8%		
3–4	10	13.9%		
**Race**				
White	64	88.9%		
Black	5	6.9%		
Other	3	4.2%		
**Smoking**				
Current	12	16.7%		
Former	54	75.0%		
Never	6	8.3%		
Pack-years			50	1.5–136
BMI			25.5	16.7–41.9
**Diabetes Mellitus**	11	15.3%		
**T-stage**				
T1a	28	38.9%		
T1b	23	31.9%		
T2a	12	16.7%		
T2b	1	1.4%		
Tx[Metastasis from another site*]	8	11.1%		
**Stage**				
IA	47	63.9%		
IB	10	13.9%		
IIA	1	1.4%		
IV	15	20.8%		
**Histology**				
Adenocarcinoma	28	38.9%		
Squamous Cell Carcinoma	16	22.2%		
NSCLC NOS	12	16.7%		
**Metastasis from other site** *	8	11.1%		
Clinical Diagnosis without biopsy	6	8.3%		
Other#	2	2.8%		
**Number of Fractions**				
3	48	66.7%		
5	24	33.3%		
**Technique**				
Conformal	58	80.5%		
VMAT	14	19.5%		

*Colorectal cancer (3), sarcoma (3), transitional cell carcinoma (1), cervical cancer (1).

# Atypical carcinoid (1), Small cell lung cancer (1).

### Tumor and dosimetric data

**Table 3** summarizes the PTV and dosimetric data. The median PTV size was 25.88 cc (range 6.71–109.44, Q1–Q3: 17.55–39.66). The median distance from the PTV to the CW was 0.00 mm (range 0–26.8, Q1–Q3: 0–3.19). The median follow up time was 20.5 months (range 9–40 months) and 29 (40.3%) patients had died by this time. The median CW V30 was 16.88 mL (range 0–88.50). The median CW maximum dose was 64.68 Gy (range 19.54–73.62). The median mEUD (a = 1) was 53.08 Gy (range 21.05–97.44).

**Table 3:** Tumor Characteristics and Dosimetry Metrics.

Characteristic	Median	Range
Distance PTV_CW [mm]	0.00	0–26.80
PTV [cc]	25.88	6.71–109.44
Maximum dose CW [Gy]	64.68	19.54–73.62
V20 [cc]	65.37	0–267.24
V30 [cc]	16.88	0–88.50
V40 [cc]	6.04	0–49.35
V50 [cc]	2.36	0–21.23
V60 [cc]	0.30	0–9.11
V70 [cc]	0	0–1.2
D(2cc) [Gy]	51.62	17.63–68.26
mEUD 1.0 [Gy]	53.08	21.05–97.44
mEUD 2.5 [Gy]	73.28	21.84–132.30
mEUD 5 [Gy]	103.73	23.10–207.85
mEUD 7.5 [Gy]	124.76	24.20–245.00
mEUD 10 [Gy]	139.96	25.15–266.64
mEUD 12.5 [Gy]	151.45	25.96–281.02
mEUD 15 [Gy]	161.40	26.66–291.41
mEUD 17.5 [Gy]	170.20	27.27–309.91
mEUD 20 [Gy]	176.56	27.80–309.91
AUC Total [Gy.cc]	9175.27	4258.02–23115.50
AUC 10 [Gy.cc]	2222.30	378–8029.37
AUC 20 [Gy.cc]	527.98	0–2634.94
AUC 30 [Gy.cc]	151.10	0–1158.37
AUC 40 [Gy.cc]	56.84	0–472.47
AUC 50 [Gy.cc]	13.06	0–172.49
AUC 60 [Gy.cc]	0.38	0–56.86
AUC 70 [Gy.cc]	0.00	0–1.39

### CW pain and/or rib fracture

A summary of CW toxicity is shown in **Table 4**. Six (8.3%) patients developed CW pain, including three (4.2%) grade 1, two (2.8%) grade 2 and one (1.4%) grade 3. Five patients developed rib fractures, including one patient who had grade 2 chest pain and a rib fracture. For the composite endpoint of any CW toxicity, which included CW pain and/or rib fracture, there were a total of 10 events (13.9%). The 1- and 2-year estimates of any CW pain were 5.6% and 11.1%, respectively. The 1- and 2-year estimates of rib fracture were 2.8% and 9.3%, respectively. The 1- and 2-year estimates of any CW toxicity were 8.4% and 18.1%, respectively. The five patients who experienced rib fractures had median maximum dose to CW of 57.37 Gy (range 54.98–71.93), median CW_V20 of 50.62 cc (range 30.96–184.47) and a median PTV of 31.05 cc (range 8.23–78.87).

**Table 4:** Incidence of Chest Wall Toxicities for 72 peripheral lung SBRT treatment plans.

Chest Wall Toxicity		N (%)	Median time to toxicty (months)	Range (months)	Q1–Q3 (months)
**Chest Wall Pain**	Grade 0	66 (91.7%)			
	Grade 1	3 (4.2%)			
	Grade 2	2 (2.8%)			
	Grade 3	1 (1.4%)			
**Any Chest Wall Pain**		6 (8.3%)	9.5	0–24	6.25–14.25
**Rib Fracture**	Asymptomatic	4 (5.6%)			
	Symptomatic*	1 (1.4%)			
**Any Rib Fracture**		5 (6.9%)	14	2–21	3–17
**Any Chest Wall Toxicity**		10 (13.9%)	9.5	0–24	3–15

*Grade 2 pain.

### Univariate and multivariate analyses of CW toxicity

On univariate analysis, none of the clinical patient characteristics (age, gender, race, diabetes, BMI, PTV distance to CW, PTV size) or dosimetric variables (CW Max, CW V20-V70, mEUD, D2cc, AUC) showed a significant association with CW pain (grade ≥ 1). P-values ranged from 0.39–1.00, well above any acceptable threshold for significance.

On multivariate analysis with forward selection, CW pain was associated with PTV volume (Odds Ratio = 1.21; 95% confidence interval: 1.01–1.46; p = 0.04), after adjusting for AUC beyond 10 Gy (or CW V20; Odds Ratio = 1.00; 95% confidence interval: 0.99–1.00; p = 0.12). However, the model was based on few events, and thus, subject to over-fitting.

### Primary Tumor Control and Local (Lobar) Control

There were 5 primary tumor failures, and the Kaplan-Meier estimate of primary tumor control at 1-year was 95.0% and at 2-years was 88.8%. There were a total of 9 primary tumor and lobar failures, and the Kaplan-Meier estimate of local control at 1-year was 90.7% and at 2-years was 83.0%;

## Discussion

We have demonstrated that a risk-adapted lung SBRT approach using 3 or 5 fractions based on CW V30 constraints resulted in a low incidence of CW toxicity (8.3%). Thus, it appears that CW toxicity from lung SBRT can be minimized with treatment planning methods, which minimize dose to the CW and incorporate a risk-adapted fractionation scheme in situations where the target size and location limit the ability to reduce the CW dose sufficiently.

Additionally, we performed a comprehensive analysis of patient and tumors characteristics and multiple dosimetric variables, including V30 [7]–[8] and mEUD [9] which have been previously associated with CW toxicity, as well as two additional metrics, D(2 ml) and AUC, which to our knowledge have not been previously studied in conjunction with risk of CW toxicity for SBRT. However, we did not demonstrate any significant associations between risk of CW injury and any of these patient, tumor or dosimetric variables. Thus, we were unable to identify additional dosimetric constraints which could further reduce the risk of CW injury in the setting of a risk-adapted 3 versus 5 fraction lung SBRT planning approach that accounts for the CW as an organ-at-risk and incorporates a V30 constraint. Lastly, neither AUC10 nor V20 show any correlation with CW toxicities.

**Table 5** shows a comparison of our results to four previous studies. The first three shown in the table, Dunlap *et al*, Woody *et al* and Stephans *et al*, all report substantially higher levels of CW toxicity than our report (18.9%–32.8% vs 8.3%). It should be noted that Woody *et al* and Stephans *et al* are both reports from the same institution, (Cleveland Clinic) though different selection criteria were used (fractionation scheme) in their respective studies. None of these three studies considered the CW as an organ-at-risk or used planning techniques to reduce the CW dose. Additionally, the majority of patients were treated with 60 Gy in 3 fractions without heterogeneity corrections with an approach comparable to that described in RTOG 0236 [6].

**Table 5:** Comparison of CW toxicity from our study versus previously reported studies.

Study	Dunlap *et al * [*7*]	Stephans *et al * [*8*]	Woody *et al * [*9*]	Lagerwaard *et al * [*10*]	Coroller and Mak *et al*
Chest wall pain (%)	32.8	22.2	18.9	12.0	8.3
Chest wall pain grade 1/2/3	2/1/17	4/6/0	6/13/1	NA	3/2/1
Number of patients	60	48	102	206	72
**Fraction x dose schemes**	3×20Gy, 5×?*	3×20Gy	3×20Gy, 4×12Gy, 5×10Gy, 10×5Gy	3×20Gy, 5×12Gy, 8×7.5Gy	3×18Gy, 5×12Gy

*Not described in the paper.

Comparatively, in the study by Lagerwaard et al [10], the CW was considered an organ-at-risk and a risk-adapted fractionation scheme was used with 20 Gy×3 for peripheral tumors and 12 Gy×5 for peripheral tumors in close proximity to the CW. With this risk-adapted fractionation schedule, the incidence of CW pain (12%) was noticeably lower than that reported for the other three series which predominantly employed a schedule of 20 Gy×3.

In comparison to the Lagerwaard et al series, which employed a risk-adapted fractionation scheme based exclusively on tumor location, we used tumor location and added a planning criterion for V30. As a result, we report an even lower incidence of CW toxicity (8.3% vs 12%) which suggests that including dosimetric factors in a risk-adapted fractionation scheme may further reduce the risk of CW injury.

The lack of heterogeneity corrections in the prior series of CW toxicity may have also played a factor in the differences in the incidence of CW toxicity observed in comparison to our study. The 20 Gy×3 fraction regimen used in RTOG 0236, and for the majority of patients in the Dunlap et al, Stephans et al and Woody et al series, is equivalent after heterogeneity correction to the 18 Gy×3 used in our series [19]. Of note, this conversion was based on a 2009 multi-institutional study which compared plans without heterogeneity corrections from patients treated on RTOG 0236 to the same plan, calculated using the same monitor units, using a superposition/convolution dose algorithm with heterogeneity corrections. This study found significant differences in dose between corrected and non-corrected plans and recommended that a lower dose (18–19 Gy for 3 fractions) be used in place of 20Gy in three fractions if heterogeneity corrections are used. This study also demonstrated that doses in normal tissues were markedly increased and exceeded the protocol-specified constraints in at least a few cases after heterogeneity correction. While CW constraints were not included in RTOG 0236, previous studies [7]–[8] have clearly indicated that the risk of CW toxicity increases rapidly if the volume receiving a high dose (e.g. V60) exceeds a given threshold. Thus, the greater accuracy of a heterogeneity corrected dose calculation may reveal high dose areas in the CW, which can potentially be reduced by plan optimization, which would not be shown if corrections were not used.

Limitations of this study include the retrospective design, and the lack of patient reported outcomes. Additionally, given the small number of CW toxicity events, our study may be underpowered to detect further associations between CW toxicity and the clinical and dosimetric variables included in our univariate analyses. Thus, additional clinical and dosimetric constraints, which may be important after controlling for V30 in the planning process and with risk-adapted fractionation, could still be identified with further study in a larger series with a larger number of total events.

In summary, risk-adapted fractionation that incorporates CW V30 constraints results in a low incidence of CW toxicity, and we did not identify any additional patient, tumor or dosimetric variables that predict for CW toxicity.

## Conclusion

Risk-adapted fractionation based on tumor and dosimetric factors can be used for lung SBRT treatments and results in a low incidence of CW toxicity. The treatment methods used at our institution have been shown to produce a lower rate of CW pain for lung SBRT patients than previously reported in the literature. A thorough analysis of patient, tumor and dosimetric variables for potential predictors of CW toxicity did not identify any statistically significant associations. We conclude that our planning methods are associated with a low risk of CW toxicity. At present, there are no apparent dosimetric parameters to pursue for further reduction of the incidence of CW pain, but with continued study, relevant parameters may be identified.
